# Improving the Selectivity of a Catalytic Film/Gas-Sensitive Film Laminated Metal Oxide Semiconductor Sensor for Mustard Using Temperature Dynamic Modulation

**DOI:** 10.3390/nano15161232

**Published:** 2025-08-12

**Authors:** Yelin Qi, Ting Liang, Wen Yang, Tengbo Ma, Siyue Zhao, Yadong Liu

**Affiliations:** Institute of NBC Defence, Beijing 102205, China

**Keywords:** MOS sensor, mustard, temperature dynamic modulation, selectivity

## Abstract

The poor selectivity of metal oxide semiconductor sensors is a major constraint to their application in the detection of chemical warfare agents. We prepared a (Pt+Pd+Rh)@Al_2_O_3_/(Pt+Rh)-WO_3_ sensor by using (Pt+Pd+Rh)@Al_2_O_3_ as a catalytic film material and (Pt+Rh)-WO_3_ as a gas-sensitive film material. Using temperature dynamic modulation, the (Pt+Pd+Rh)@Al_2_O_3_/(Pt+Rh)-WO_3_ sensor was realised to improve the selectivity for mustard. Due to the catalytic effect of the (Pt+Pd+Rh)@Al_2_O_3_ catalytic film on mustard, mustard was able to be catalytically generated into mustard sulphoxide after passing through the (Pt+Pd+Rh)@Al_2_O_3_ catalytic film. Under a certain temperature dynamic modulation, the mustard concentration on the surface of the (Pt+Rh)-WO_3_ gas-sensitive film showed an increase and then a decrease. Since the resistance response of the (Pt+Rh)-WO_3_ gas-sensitive film to mustard was much higher than that of mustard sulphoxide, the change in the resistance of the (Pt+Rh)-WO_3_ gas-sensitive film was mainly determined by the change in the concentration of mustard, which led to the peak signal in the curve of its resistance response to mustard. The experimental results showed that the (Pt+Pd+Rh)@Al_2_O_3_/(Pt+Rh)-WO_3_ sensor had peak signals in the resistance response to mustard only, and not in the resistance response to 12 interfering gases, such as carbon monoxide.

## 1. Introduction

Metal oxide semiconductor (MOS) sensors have the advantages of good sensitivity, response speed, service life, etc. [1,2,3,4,5], and have promising applications in the field of chemical warfare agent detection. However, there are still technical difficulties with the poor selectivity of MOS sensors for chemical warfare agents [6]. Therefore, improving the selectivity of MOS sensors for chemical warfare agents is of great value.

Various methods have been used to improve the selectivity of MOS sensors for chemical warfare agents, e.g., noble metal doping [7], construction of heterostructures [8], variable-temperature heating [9], etc. Niu [10] used SnO_2_ nanosheets as the sensing material for MOS sensors and tested the transient resistance of the MOS sensors to ethanol, formaldehyde, toluene, and acetone in the temperature pulse operating mode; extracted the dynamic characteristics of the resistance response of each of them; and was able to accurately identify four gases above 1 ppm. Yang [11] prepared Au-O-SnO_2_ by doping Au nanoparticles (NPs) into SnO_2_ with oxygen-rich vacancies, which showed high selectivity toward sarin (DMMP) under the interference of five gases, including ethanol. This was attributed to the electron sensitisation effect of Au NPs, which led to the formation of a Schottky junction between Au and SnO_2_, thus improving the overall response to DMMP. Poloju [12] prepared ZnO/CuO nanocomposites by the sol–gel method, and the ZnO/CuO nanocomposites showed better gas sensitivity to NH3 at room temperature compared with pure ZnO nanoparticles. This was due to the fact that the ZnO/CuO nanocomposites formed an electron depletion layer at the p-n heterojunction on the surface of ZnO and CuO, and the depleted holes in CuO were quickly replenished from the electron depletion layer at the p-n heterojunction, which, in turn, enhanced the response to NH_3_.

By compiling and analysing the literature [13,14,15,16,17,18,19,20], we concluded that simultaneous improvements in the materials, structure, and operating conditions of MOS sensors could effectively improve the selectivity of the sensors for chemical warfare agents. Based on the method of this selectivity improvement, we successfully achieved the high selectivity of a CeMnOx/Pt@SnO_2_ sensor for hydrocyanic acid under a certain temperature dynamic modulation in our previous work. Meanwhile, the resistance response of the MOS sensor to hydrocyanic acid also showed a good response time, stability, and anti-interference, which verified the feasibility of such a selectivity improvement method [21].

In this paper, we systematically propose a method to improve the selectivity of MOS sensors for mustard (dichlorodiethyl sulphide, abbreviated as HD), a vesicant chemical warfare agent. Firstly, we screened gas-sensitive film materials and catalytic film materials with good sensing performance and made the materials into a laminated MOS sensor, among which (Pt+Rh)-WO_3_ showed a good resistance response to HD, and (Pt+Pd+Rh)@Al_2_O_3_ showed a good catalytic ability against HD. Then, there were peak signals in the resistance response of the (Pt+Pd+Rh)@Al_2_O_3_/(Pt+Rh)-WO_3_ sensor for HD under temperature dynamic modulation, and the specificity of the signals indicated that the MOS sensor possessed high selectivity for HD. Finally, we investigated the sensing mechanism of selectivity improvement and analysed the reasons for the generation of specific peak signals.

## 2. Materials and Methods

### 2.1. Preparation of Layer Materials

All chemical reagents used for material preparation in this paper were purchased from Aladdin Reagent Co., Ltd., Shanghai, China.

#### 2.1.1. Preparation of (Pt+Rh)-WO_3_ Slurry

The precursor was obtained by dispersing 10 mmol of WCl_6_ in 50 mL of C_7_H_16_ and stirring for 1 h. The reaction was carried out at 160 °C for 24 h. After drying, the precursor was washed twice with C_2_H_6_O, CH_2_Cl_2_, and purified water, respectively, and then placed in an oven and burned at 450 °C for 2 h to obtain WO_3_ matrix powder. An amount of 2 g of WO_3_ powder dispersed in 20 mL of C_2_H_6_O, the calculated 0.01 g/mL H_2_PtCl_6_-6H_2_O ionic solution, and the 0.01 g/mL RhCl_3_-3H_2_O ionic solution were added and stirred for 30 min. Then, the mixture was put into an oven and dried at 60 °C to obtain 0.5 wt% Pt^4+^, Rh^3+^-modified WO_3_ powder. The modified WO_3_ powder was obtained using the above steps. An amount of 3 g of (Pt+Rh)-WO_3_ powder was added to 9 mL of organic slurry and ball-milled at 400 rpm for 4 h to obtain (Pt+Rh)-WO_3_ slurry.

#### 2.1.2. Preparation of (Pt+Pd+Rh)@Al_2_O_3_ Slurry

According to the ratio of Pt (Pd, Rh): Al_2_O_3_, 1 g of γ-Al_2_O_3_ and the calculated 0.5 wt% 0.01 g/mL H_2_PtCl_6_-6H_2_O ionic solution, 0.5 wt% 0.01 g/mL PdCl_2_-2H_2_O ionic solution, and 0.5 wt% 0.01 g/mL of RhCl_3_-3H_2_O ionic solution were added to a ball milling jar and ball milled at 400 rpm for 4 h. After drying, the powder was ground to obtain (Pt+Pd+Rh)@Al_2_O_3_ powder. Then, 3 g of (Pt+Pd+Rh)@Al_2_O_3_ powder was added to 9 mL of organic slurry to obtain (Pt+Pd+Rh)@Al_2_O_3_ powder slurry.

### 2.2. Preparation of the Catalytic Layer/Gas-Sensitive Layer MOS Sensor

**Figure 1:**
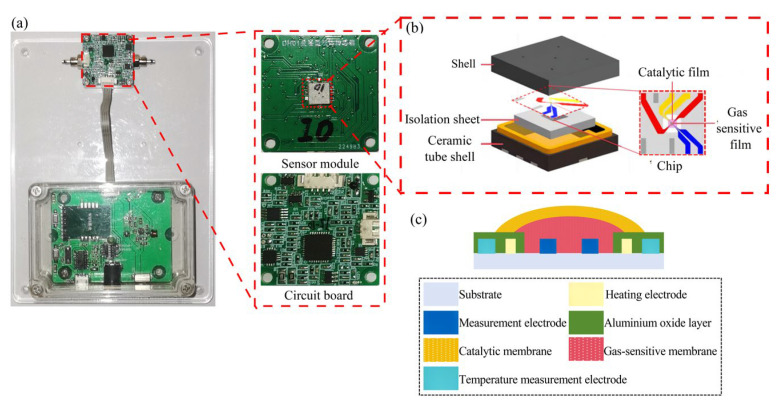
(**a**) The physical diagram of the MOS sensor; (**b**) the sensing module; (**c**) the design of the sensor chip [21].

Figure 1a shows the testing module and transducer part of the MOS sensor. The gas-sensitive module was contained inside the sensor part, as shown in Figure 1b. The gas-sensitive module consisted of a shell, an isolation sheet, a ceramic tube shell and a chip, and the electrode area (50 × 50 μm) of the chip was covered by the gas-sensitive film and catalytic film. Figure 1c shows the structural design of the chip, in which zirconia was used as the substrate material to ensure that the substrate had good mechanical strength. The heating electrode and temperature measurement electrode materials were made of Pt. This is due to the fact that Pt has good electrical conductivity and a stable resistance-temperature coefficient, which enables it to conduct current efficiently, with a linear relationship between resistance and temperature. The measurement electrode material was made of Au, which had lower resistivity and could obtain the resistance change in the MOS material more efficiently. The aluminium oxide layer was covered on the surface of the heating electrode and the measuring electrode to isolate the two electrodes because aluminium oxide has high resistance, making it suitable as an insulating material and preventing circuit short-circuiting when the electrode was sprayed with film.

### 2.3. Heating Mode

**Figure 2:**
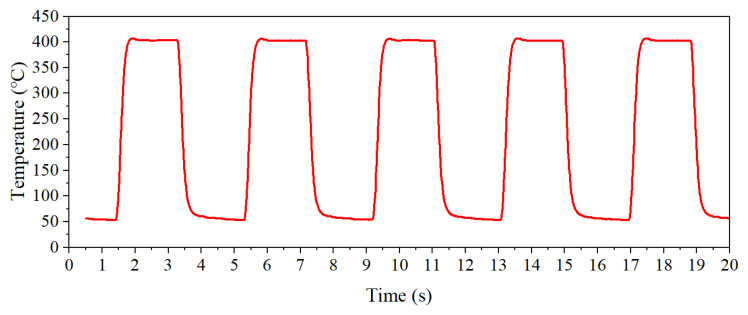
The rectangular wave heating mode.

In this paper, the rectangular wave heating mode was adopted, and its heating process is shown in Figure 2. The default heating parameters were T1 = 400 °C, T2 = 100 °C, t1 = 1 s, and t2 = 1 s (T1 represents the high temperature set value, T2 represents the low temperature set value, t1 represents the high temperature holding time, and t2 represents the low temperature holding time).

## 3. Results and Discussion

### 3.1. Characterisation of Material

**Figure 3:**
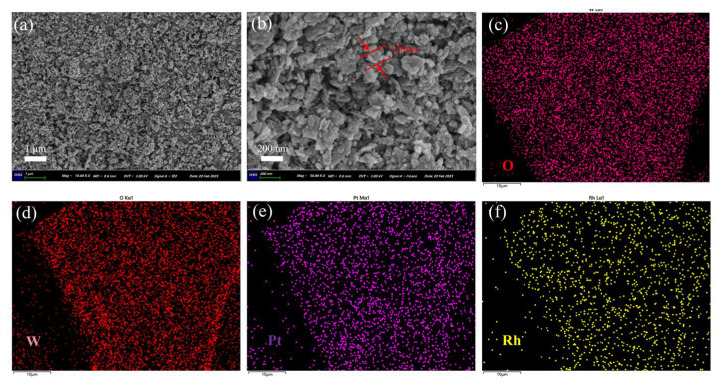
(**a**,**b**) SEM and (**c**–**f**) EDS images of (Pt+Rh)-WO_3_ powder.

Figure 3 shows the SEM image of (Pt+Rh)-WO_3_ powder. The structure of (Pt+Rh)-WO_3_ was composed of many nanoparticles and nanosheets stacked together, and the diameter of the synthesised (Pt+Rh)-WO_3_ nanoparticles was about 120 nm. The material had a large specific surface area, which could provide more adsorption sites, and helped to enhance the adsorption efficiency and the reaction rate of HD, as shown in Figure 3a,b. The major elements W, O, Pt and Rh were uniformly distributed within the (Pt+Rh)-WO_3_ powder, as shown in Figure 3c–f.

**Figure 4:**
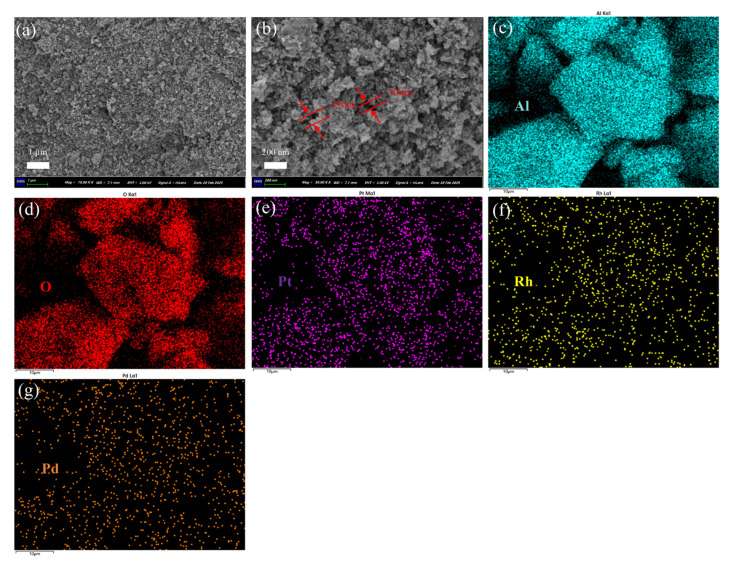
(**a**,**b**) SEM and (**c**–**g**) EDS images of (Pt+Pd+Rh)@Al_2_O_3_ powder.

Figure 4 shows the SEM image of (Pt+Pd+Rh)@Al_2_O_3_ powder. The powder did not show a clear fixed morphology and appeared to be made up of a multitude of nanoparticles stacked together, as shown in Figure 4a,b. The major elements Al, O, Pt, Rh and Pd were uniformly distributed within the (Pt+Pd+Rh)@Al_2_O_3_ powder, as shown in Figure 4c–g.

**Figure 5:**
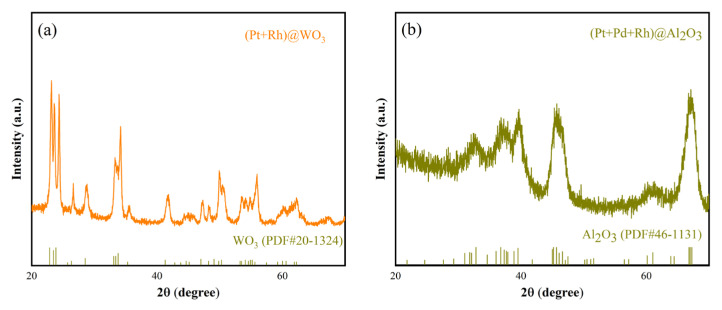
XRD patterns of (**a**) (Pt+Rh)-WO_3_ and (**b**) (Pt+Pd+Rh)@Al_2_O_3_.

The XRD patterns of (Pt+Rh)-WO_3_ and (Pt+Pd+Rh)@Al_2_O_3_ are shown in Figure 5a,b, respectively. The patterns show that both (Pt+Rh)-WO_3_ and (Pt+Pd+Rh)@Al_2_O_3_ are of high purity with almost no additional impurity peaks. WO_3_ can be indexed according to the corresponding standard card (20-1324) and Al_2_O_3_ can be indexed according to the corresponding standard card (46-1131), but due to the small amount of modification of Pt^4+^, Rh^3+^, and Pd^2+^, it is difficult to observe the relevant diffraction information of the these modified elements from the patterns.

### 3.2. The Peak Signal and Optimisation of the Heating Parameters

**Figure 6:**
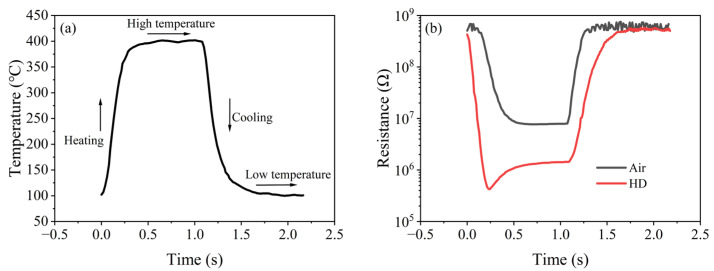
Single-cycle resistance response of the (Pt+Pd+Rh)@Al_2_O_3_/(Pt+Rh)-WO_3_ sensor to air and HD under rectangular wave heating mode: (**a**) temperature change; (**b**) resistance change in air and HD.

Figure 6a shows the single-cycle temperature–time (T-t) curve of the rectangular wave heating mode. The temperature noise of the gas-sensitive chip was ±5 °C at 400 °C and ±4 °C at 100 °C, and the single-cycle time was about 2.2 s, indicating that the sensor had an accurate and stable rectangular wave heating capability.

Figure 6b shows the single-cycle resistance–time (R-t) curve of air and HD. As the temperature of the sensor changed, a negative correlation in resistance ensued. According to the theory of grain-boundary barrier control [22], the increase in temperature caused the internal grain-boundary barrier of (Pt+Rh)-WO_3_ to decrease, which weakened the hindering effect on the free electrons and led to the decrease in the resistance of (Pt+Rh)-WO_3_. The resistance of the sensor was reduced in HD atmosphere compared to that in air atmosphere. According to the adsorption/desorption model, this was because (Pt+Rh)-WO_3_, as an N-type semiconductor, exhibited a decreasing trend in resistance when interacting with reducing gases such as HD compared to in air.

It is noteworthy that a minimum in the resistance response to the HD was observed near 323 °C in the heating interval, which manifested as a distinct peak signal.

In order to quantify the peak signal of HD, a reasonable calculation method needed to be constructed. By analysing the data and converting the T-t data and R-t data into conductance–temperature (G-T) data as a peak curve—where the conductance is the reciprocal of the resistance—the peak height of the peak signal can be reasonably quantified.

**Figure 7:**
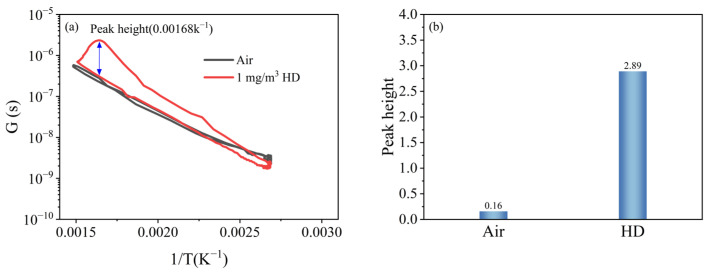
Peak signals of HD and air: (**a**) peak curves; (**b**) peak heights.

Figure 7a shows the peak curves of air and 1 mg/m^3^ HD. It can be seen that the curve of 1 mg/m^3^ HD had a prominent peak, so the height of this peak can be used to quantify the peak signal.

The optimal catalytic temperature of the catalytic film for HD is Tc, which corresponds to the highest point of the peak of the G-T curve. The peak height of HD, S_Gas_, is defined as the ratio of the conductance at the top of the peak in the G-T curve to that at the bottom of the peak at the corresponding temperature, and it can be calculated as

(1) $$S_{\mathrm{Gas}}=\frac{R_{\left(\mathrm{Gas},H,\mathrm{Tc}\right)}^{-1}}{R_{\left(\mathrm{Gas},C,\mathrm{Tc}\right)}^{-1}}=\frac{R_{\left(\mathrm{Gas},C,\mathrm{Tc}\right)}}{R_{\left(\mathrm{Gas},H,\mathrm{Tc}\right)}}$$

where R_(Gas, C, Tc)_ and R^−1^_(Gas, C, Tc)_ denote the resistance when cooling to Tc and the conductance in HD atmosphere, respectively, and R_(Gas, H, Tc)_, R^−1^_(Gas, H, Tc)_ denote the resistance when heating to Tc and the conductance of the corresponding resistance. Similarly, the peak height S_Air_ of air is

(2) $$S_{\mathrm{Air}}=\frac{R_{\left(\mathrm{Air},H,\mathrm{Tc}\right)}^{-1}}{R_{\left(\mathrm{Air},C,\mathrm{Tc}\right)}^{-1}}=\frac{R_{\left(\mathrm{Air},C,\mathrm{Tc}\right)}}{R_{\left(\mathrm{Air},H,\mathrm{Tc}\right)}}$$

where R_(Air, C, Tc)_, R^−1^_(Air, C, Tc)_ denote the resistance when cooling to Tc and the conductance in air atmosphere, respectively, and R_(Air, H, Tc)_, R^−1^_(Air, H, Tc)_ denote the resistance when heating to Tc and the conductance of the corresponding resistance. Therefore, the peak height S_Peak_ of HD is

(3) $$S_{\mathrm{Peak}}{=S}_{\mathrm{Gas}}-S_{\mathrm{Air}}=\frac{R_{\left(\mathrm{Gas},C,\mathrm{Tc}\right)}}{R_{\left(\mathrm{Gas},H,\mathrm{Tc}\right)}}-\frac{R_{\left(\mathrm{Air},C,\mathrm{Tc}\right)}}{R_{\left(\mathrm{Aiv},H,\mathrm{Tc}\right)}}$$

Following the quantification method described above, the peak height of 1 mg/m^3^ HD was calculated to be 2.89, and the intrinsic peak height of air was 0.16, as shown in Figure 7b.

To further enhance the peak signal of HD, the heating parameters needed to be optimised, and the optimised heating parameters were used as test parameters. The heating parameters in the rectangular wave heating mode were set within a range gradient, and the resistance response of the (Pt+Pd+Rh)@Al_2_O_3_/(Pt+Rh)-WO_3_ sensor to 1 mg/m^3^ HD was tested under the heating parameter gradient. The resistance response data were collated into peak curves, and their peak heights were calculated.

**Figure 8:**
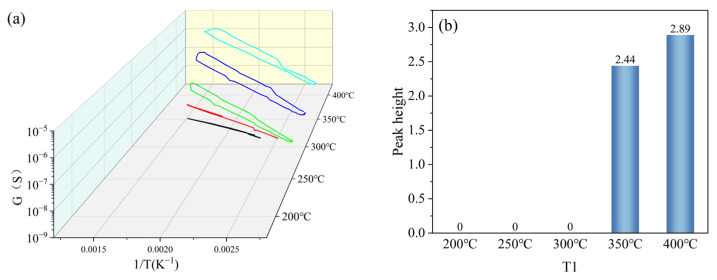
Peak signals of HD at T1 gradient: (**a**) peak curves; (**b**) peak heights.

T1 was set with a gradient of 200 °C, 250 °C, 300 °C, 350 °C, and 400 °C (the colours were black, red, green, blue and cyan, in that order), and other parameters were kept constant (T2 = 100 °C, t1 = 1 s, t2 = 1 s). Under the gradient set for T1, the peak signal of the peak curve became more obvious as the set temperature value of T1 increased, as shown in Figure 8a. When T1 = 200 °C, 250 °C, and 300 °C, the peak height was 0. When T1 = 350 °C and 400 °C, the peak height was greater than 0, and the peak height at T1 = 400 °C was greater than that at T1 = 350 °C, as shown in Figure 8b. Based on the peak heights of the peak curves under the T1 gradient, T1 in the test parameters was set to 400 °C.

**Figure 9:**
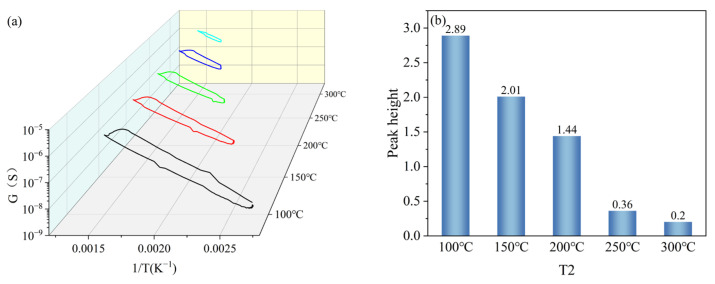
Peak signals of HD at T2 gradient: (**a**) peak curves; (**b**) peak heights.

T2 was set with a gradient of 100 °C, 150 °C, 200 °C, 250 °C, and 300 °C, and other parameters were kept constant (T1 = 400 °C, t1 = 1 s, t2 = 1 s). Under the gradient set for T2, the peak signals of the peak curves became less obvious as the set temperature value of T2 increased, as shown in Figure 9a. The peak heights were 2.89, 2.01, 1.44, 0.36, and 0.2 when the set temperature values of T2 were 100 °C, 150 °C, 200 °C, 250 °C, and 300 °C, respectively, and the peak heights gradually decreased with the increase in the set temperature values of T2, as shown in Figure 9b. Based on the peak heights of the peak curves under the T2 gradient, the T2 in the test parameters was set to 100 °C.

**Figure 10:**
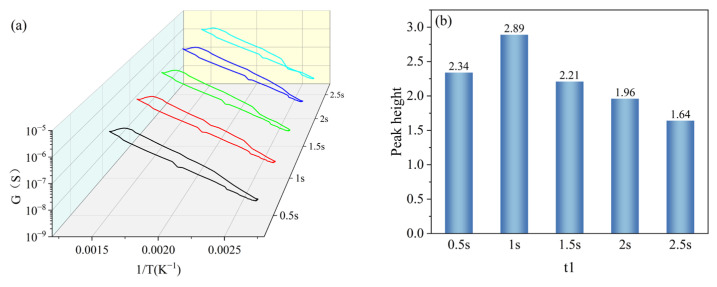
Peak signals of HD at t1 gradient: (**a**) peak curves; (**b**) peak heights.

t1 was set with a gradient of 0.5 s, 1 s, 1.5 s, 2 s, and 2.5 s, and other parameters were kept constant (T1 = 400 °C, T2 = 100 °C, t2 = 1 s). Under the gradient set for t1, the peak signals appeared in all peak curves, and the peak heights were 2.34, 2.89, 2.21, 1.96, and 1.64, respectively, as shown in Figure 10a. And with the increase in the set holding time of t1, the peak heights were increased firstly and then decreased, and reached the maximum value at t1 = 1 s, as shown in Figure 10b. Based on the peak heights of the peak curves under the t1 gradient, t1 in the test parameters was set to 1 s.

**Figure 11:**
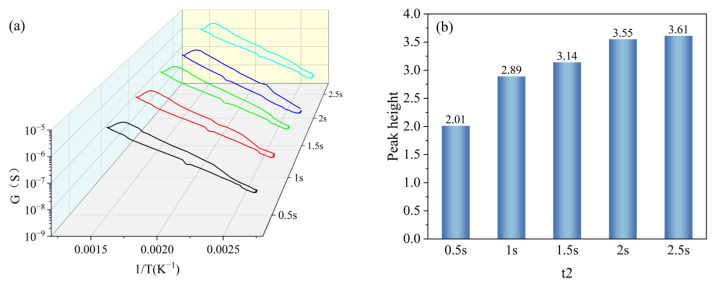
Peak signals of HD at t2 gradient: (**a**) peak curves; (**b**) peak heights.

t2 was set with a gradient of 0.5 s, 1 s, 1.5 s, 2 s, and 2.5 s, and other parameters were kept constant (T1 = 400 °C, T2 = 100 °C, t1 = 1 s). Under the gradient set for t2, the peak signals appeared in all peak curves, and the peak heights were 2.01, 2.89, 3.14, 3.55, and 3.61, respectively, as shown in Figure 11a. And the peak heights increased gradually with the increase in the set holding time of t2 and reached the maximum value at t2 = 2.5 s, as shown in Figure 11b. The peak height at t2 = 2.5 s increased less compared to that at t2 = 2 s; however, the increase in the single-cycle time was larger, so it was more reasonable to set t1 as 2 s.

Based on the peak heights of the 1 mg/m^3^ HD resistance response under the optimisation of the heating parameters described above, and taking into account the single-cycle test time, the test parameters for HD were determined to be T1 = 400 °C, T2 = 100 °C, t1 = 1 s, and t2 = 2 s.

### 3.3. Selectivity Testing

The specificity of the HD peak signal was verified by comparing the peak heights of the 1 mg/m^3^ HD and interfering gases' peak curves under the test parameters. The interfering gases were purchased from Clean Energy Technology Co., Ltd. (Fushun, China) at a concentration of 25 ppm, with nitrogen as the carrier gas.

**Figure 12:**
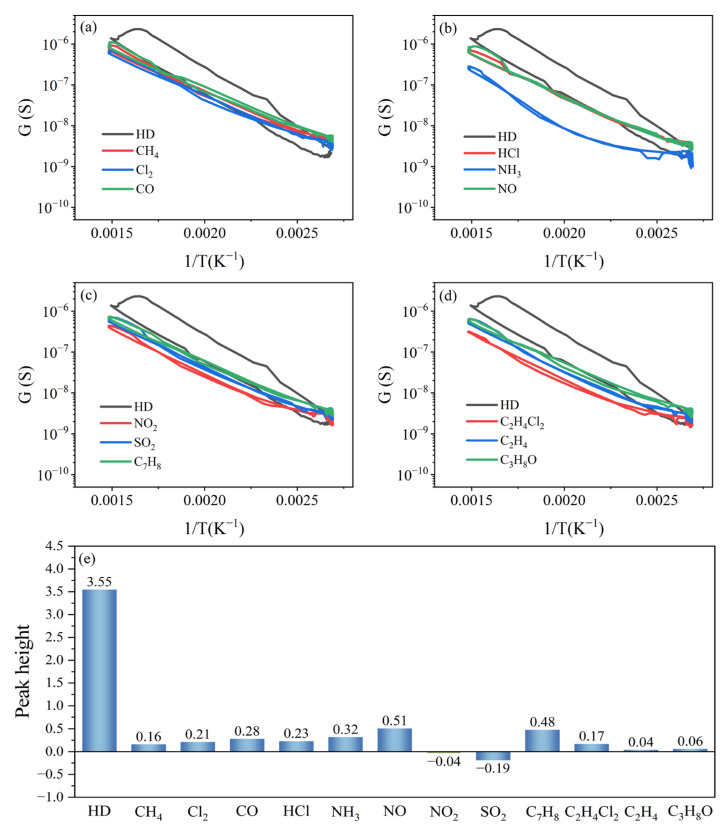
(**a**–**d**) Peak curves and (**e**) peak heights of HD and interfering gases.

Compared to the peak curves of the interfering gases, there was a distinct peak signal in the peak curve of HD only, as shown in Figure 12a–d.

The peak heights of HD, CH_4_, Cl_2_, CO, HCl, NH_3_, NO, NO_2_, SO_2_, C_7_H_8_, C_2_H_4_Cl_2_, C_2_H_4_, and C_3_H_8_O were 3.55, 0.16, 0.21, 0.28, 0.23, 0.32, 0.51, −0.04, −0.19, 0.48, 0.17, 0.04, and 0.06, as shown in Figure 12e. This shows that the peak height of HD was much larger than that of the interfering gases, the peak signal of HD was specific, and the (Pt+Pd+Rh)@Al_2_O_3_/(Pt+Rh)-WO_3_ sensor had high selectivity for HD under the test parameters.

## 4. Sensing Mechanism

In order to systematically investigate the sensing mechanism by which the (Pt+Pd+Rh)@Al_2_O_3_/(Pt+Rh)-WO_3_ sensor achieved high selectivity for HD under temperature dynamic modulation, we explored three aspects: the catalytic mechanism, the gas-sensitive mechanism, and the synergistic mechanism between the catalytic film and gas-sensitive film.

### 4.1. Catalytic Mechanism

**Figure 13:**
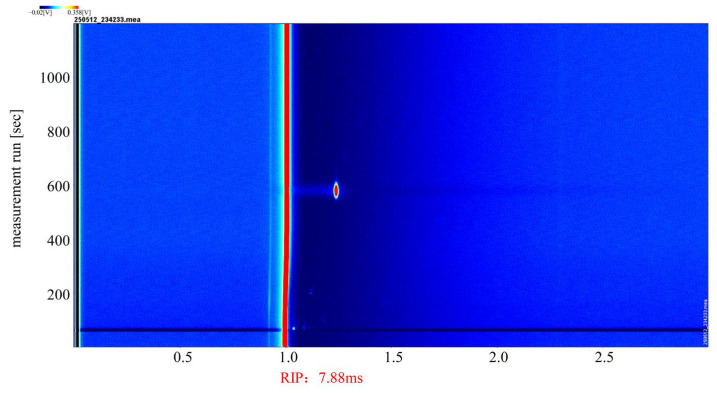
GC-IMS profile of HD.

The catalytic effect of the (Pt+Pd+Rh)@Al_2_O_3_ catalytic film on HD was analysed. Figure 13 shows the GC-IMS profile of HD in positive ion mode, which served as a reference for the GC-IMS profile of its catalytic products.

**Figure 14:**
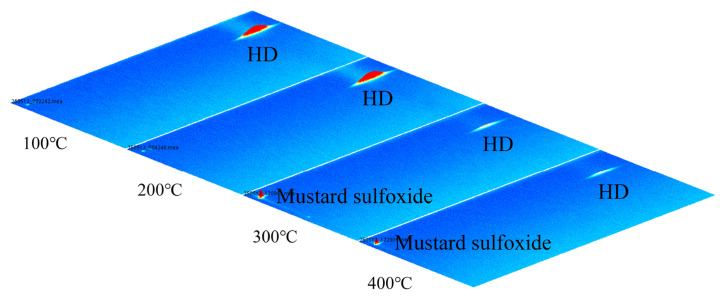
GC-IMS profile of HD-catalysed products.

When the temperatures of the (Pt+Pd+Rh)@Al_2_O_3_ catalytic film were 100 °C and 200 °C, qualitative analysis via GC-IMS showed that the catalytic product remained HD; when the temperatures of (Pt+Pd+Rh)@Al_2_O_3_ catalytic film were 300 °C and 400 °C, the catalytic product was mustard sulphoxide, as shown in Figure 14. This indicates that the (Pt+Pd+Rh)@Al_2_O_3_ catalytic film did not catalyse HD at 100 °C and 200 °C, but exhibited a more efficient catalytic effect on HD at 300 °C and 400 °C.

**Figure 15:**
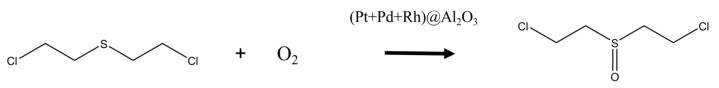
Catalytic oxidation of HD in the presence of (Pt+Pd+Rh)@Al_2_O_3_ catalytic film.

Based on the qualitative results of GC-IMS, it was speculated that when the temperature of the (Pt+Pd+Rh)@Al_2_O_3_ catalytic film reached above the optimum catalytic temperature for HD, a catalytic oxidation reaction took place as HD passed through the (Pt+Pd+Rh)@Al_2_O_3_ catalytic film, producing mustard sulphoxide and water, as shown in Figure 15.

The catalytic effects of the (Pt+Pd+Rh)@Al_2_O_3_ catalytic film on the interfering gases were analysed. Table 1 summarises the catalytic products of HD and 12 interfering gases after passing through the (Pt+Pd+Rh)@Al_2_O_3_ catalytic film at different temperatures.

Based on the analytical approach to catalysis described above, the catalytic products of interfering gases were characterised, and it was found that the catalysis of interfering gases by the (Pt+Pd+Rh)@Al_2_O_3_ catalytic films can be classified into three types:

The first type was that the (Pt+Pd+Rh)@Al_2_O_3_ catalytic film did not catalyse interfering gases under heating conditions, including CO, NO_2_, C_2_H_4_, SO_2_, C_2_H_4_Cl_2_, NH_3_, HCl and Cl_2_. In the case of catalysis of HCl, for example, the concentration of HCl did not decrease, and no other AIP signals were captured when the catalytic film was heated at 100 °C, 200 °C, 300 °C and 400 °C, indicating that no catalytic reaction occurred when HCl passed through the catalytic film.

The second type was that the (Pt+Pd+Rh)@Al_2_O_3_ catalytic film catalysed interfering gases under heating conditions, including NO and C_3_H_8_O. In the case of C_3_H_8_O, it was converted to C_5_H_8_O_3_ by the catalytic film at 100 °C, 200 °C, 300 °C and 400 °C.

The third type was that the (Pt+Pd+Rh)@Al_2_O_3_ catalytic film catalysed interfering gases under heating conditions, including C_7_H_8_. Similarly to the catalysis of HD, the (Pt+Pd+Rh)@Al_2_O_3_ catalytic film did not catalyse C_7_H_8_ at 100 °C, but was able to catalyse C_7_H_8_ to C_7_H_6_O at 200 °C, 300 °C and 400 °C.

### 4.2. Gas-Sensitive Mechanism

The gas-sensitive mechanism of the (Pt+Rh)-WO_3_ gas-sensitive film on HD was analysed based on the oxygen adsorption model [23]. The interaction between HD and reactive oxygen species in the (Pt+Rh)-WO_3_ lattice was as follows:

(4) $$O_{2}^{-}+2S{\left({\mathrm{ClCH}}_{2}{\mathrm{CH}}_{2}\right)}_{2}=2S{\left({\mathrm{ClCH}}_{2}{\mathrm{CH}}_{2}\right)}_{2}{O+e}^{-}$$

(5) $$O^{-}+S{\left({\mathrm{ClCH}}_{2}{\mathrm{CH}}_{2}\right)}_{2}=S{\left({\mathrm{ClCH}}_{2}{\mathrm{CH}}_{2}\right)}_{2}{O+e}^{-}$$

(6) $$O^{2-}+S{\left({\mathrm{ClCH}}_{2}{\mathrm{CH}}_{2}\right)}_{2}=S{\left({\mathrm{ClCH}}_{2}{\mathrm{CH}}_{2}\right)}_{2}{O+2e}^{-}$$

When HD was in contact with the (Pt+Rh)-WO_3_ gas-sensitive film, it was adsorbed at the Pt+Rh active sites, releasing electrons that were injected into the WO_3_ conduction band. This caused the electron depletion layer on the surface of the gas-sensitive film to be contracted, and the resistance to carrier migration would be reduced, resulting in a reduction in the resistance of the gas-sensitive film.

**Figure 16:**
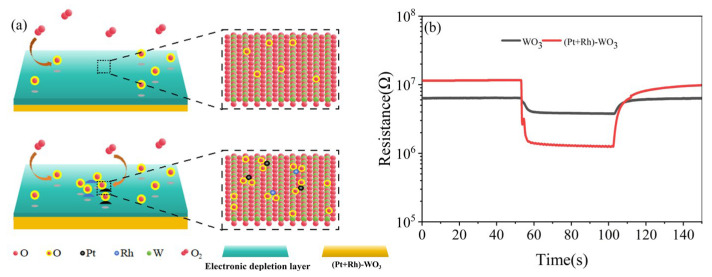
(**a**) Sensitivity of WO_3_ and (Pt+Rh)-WO_3_ gas-sensitive film to HD; (**b**) resistance response of WO_3_ and (Pt+Rh)-WO_3_ gas-sensitive film to 1 mg/m^3^ of HD at 400 °C.

Pt+Rh doping had a synergistic effect on the modulation of the WO_3_ gas-sensitive film: on the one hand, it generated ligand-unsaturated active sites on its surface, inhibited agglomeration, and achieved uniform dispersion, thereby increasing the number of active sites, lowering the adsorption barriers of HD and enhanced the rate; on the other hand, the complementary electronic structure of Pt+Rh optimised the adsorption of oxygen species, promoted the dissociation of O_2_ into highly reactive oxygen species, and increased the probability of oxidation reactions with HD, as shown in Figure 16a. By comparing the resistance responses of the WO_3_ gas-sensitive film and the (Pt+Rh)-WO_3_ gas-sensitive film to 1 mg/m^3^ HD at 400 °C, the sensitivity ((Ra-Rg)/Ra) increased from 38% to 89%, as shown in Figure 16b.

**Figure 17:**
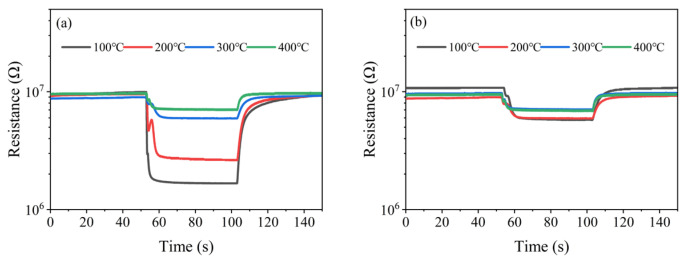
Resistance response of (Pt+Rh)-WO_3_ gas-sensitive film to (**a**) HD and (**b**) toluene catalytic products.

Figure 17a,b show the resistance responses of the (Pt+Rh)-WO_3_ gas-sensitive film to HD and toluene catalytic products at different temperatures of the (Pt+Pd+Rh)@Al_2_O_3_ catalytic film, respectively. When the temperature of the (Pt+Pd+Rh)@Al_2_O_3_ catalytic film was increased from 100 °C and 200 °C to 300 °C and 400 °C, the sensitivity of the (Pt+Rh)-WO_3_ gas-sensitive film to HD catalytic products decreased significantly. In combination with the results of the catalytic mechanism study, this indicated that the resistance response of the (Pt+Rh)-WO_3_ gas-sensitive film to HD was significantly higher than that to mustard sulphoxide. However, when the temperature of the (Pt+Pd+Rh)@Al_2_O_3_ catalytic film was changed, the sensitivity of the (Pt+Rh)-WO_3_ gas-sensitive film to the toluene catalytic product did not show a significant change, suggesting that the (Pt+Rh)-WO_3_ gas-sensitive film had similar resistance responses to toluene and benzaldehyde.

### 4.3. Synergistic Mechanism Between the Catalytic Film and Gas-Sensitive Film

**Figure 18:**
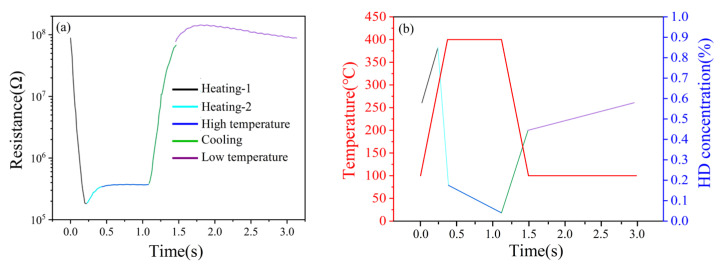
(**a**) Single-cycle resistance response of HD; (**b**) simulation of the variation in HD gas concentration on the surface of (Pt+Rh)-WO_3_ gas-sensitive film.

The single cycle was divided into five intervals, i.e., heating-1, heating-2, high temperature, cooling and low temperature, as shown in Figure 18a. Since the HD peak signals appeared in the three intervals of heating-1, heating-2 and high temperature, the analysis focused on the synergistic mechanism of the (Pt+Pd+Rh)@Al_2_O_3_ catalytic film and the (Pt+Rh)-WO_3_ gas-sensitive film on HD in these three intervals.

For the heating-1 interval, as the temperature increased, the (Pt+Pd+Rh)@Al_2_O_3_ catalytic film did not reach the optimal catalytic temperature for HD, while the adsorption capacity of the (Pt+Rh)-WO_3_ gas-sensitive film for HD was gradually enhanced. This led to a gradual increase in the concentration of HD on the surface of the (Pt+Rh)-WO_3_ gas-sensitive film. Due to the high resistance response of the (Pt+Rh)-WO_3_ gas-sensitive film to HD, the resistance decreased rapidly.

For the heating-2 interval, as the temperature continued to increase, the (Pt+Pd+Rh)@Al_2_O_3_ catalytic film reached the optimum catalytic temperature for HD. At this time, the stronger catalytic ability of the (Pt+Pd+Rh)@Al_2_O_3_ catalytic film for HD led to a rapid decrease in the concentration of HD on the surface of the (Pt+Rh)-WO_3_ gas-sensitive film and a rapid increase in the concentration of catalytic products. However, the resistance response of the (Pt+Rh)-WO_3_ gas-sensitive film to the catalytic product was lower than that to HD, and thus the resistance showed a rapid recovery, i.e., the appearance of the peak signal.

For the high temperature interval, the (Pt+Pd+Rh)@Al_2_O_3_ catalytic film still had strong catalytic ability for HD, while the (Pt+Rh)-WO_3_ gas-sensitive film had a high resistance response to HD. Due to the continuous catalysis of HD by the (Pt+Pd+Rh)@Al_2_O_3_ catalytic film, the concentration of HD on the surface of the (Pt+Rh)-WO_3_ gas-sensitive film decreased slowly, and thus the resistance showed a slow increase.

Figure 18b shows the simulation results of the variation in HD concentration on the surface of the (Pt+Rh)-WO_3_ gas-sensitive film during a single cycle.

## 5. Conclusions

In this paper, a method to achieve high selectivity of MOS sensors for HD based on specific peak signals was presented. Under a certain temperature dynamic modulation, the resistance response of the (Pt+Pd+Rh)@Al_2_O_3_/(Pt+Rh)-WO_3_ sensor to HD showed one peak signal; however, it did not exist in the resistance response to the interfering gas. This was due to the sensitivity of the WO_3_ gas-sensitive to HD was significantly enhanced by the modification of Pt and Rh, resulting in an increase in the resistance response of the (Pt+Rh)-WO_3_ gas-sensitive film to mustard from 38% to 89%. And the ability of the (Pt+Pd+Rh)@Al_2_O_3_ catalytic film to catalyse the formation of mustard sulphoxide from HD after reaching the optimum catalytic temperature for HD. During the heating interval of the catalytic film, the concentration of HD on the surface of the (Pt+Rh)-WO_3_ gas-sensitive film first increased and then decreased. Owing to the stronger resistance response of the gas-sensitive film to HD than to mustard sulphoxide, specific peak signals were generated in the resistance response.

## Figures and Tables

**Table 1:** Catalytic products of HD and interfering gases.

Gases	Catalytic Products (Based on AIP Signals in GC-IMS Profiles)			
	100 °C	200 °C	300 °C	400 °C
HD	HD	HD	HD, C_4_H_8_Cl_2_OS	HD, C_4_H_8_Cl_2_OS
CO	CO	CO	CO	CO
NO	NO, NO_2_	NO, NO_2_	NO, NO_2_	NO, NO_2_
NO_2_	NO_2_	NO_2_	NO_2_	NO_2_
CH_4_	-	-	-	-
C_2_H_4_	C_2_H_4_	C_2_H_4_	C_2_H_4_	C_2_H_4_
SO_2_	SO_2_	SO_2_	SO_2_	SO_2_
C_7_H_8_	C_7_H_8_	C_7_H_8_, C_7_H_6_O	C_7_H_8_, C_7_H_6_O	C_7_H_8_, C_7_H_6_O
C_2_H_4_Cl_2_	C_2_H_4_Cl_2_	C_2_H_4_Cl_2_	C_2_H_4_Cl_2_	C_2_H_4_Cl_2_
NH_3_	NH_3_	NH_3_	NH_3_	NH_3_
HCl	HCl	HCl	HCl	HCl
Cl_2_	Cl_2_	Cl_2_	Cl_2_	Cl_2_
C_3_H_8_O	C_3_H_8_O, C_5_H_8_O_3_	C_3_H_8_O, C_5_H_8_O_3_	C_3_H_8_O, C_5_H_8_O_3_	C_3_H_8_O, C_5_H_8_O_3_

## Data Availability

Data are contained within the article.
